# Isokinetic Leg Strength and Power in Elite Handball Players

**DOI:** 10.2478/hukin-2014-0050

**Published:** 2014-07-08

**Authors:** José M. González-Ravé, Daniel Juárez, Jacobo A. Rubio-Arias, Vicente J Clemente-Suarez, María A Martinez-Valencia, Javier Abian-Vicen

**Affiliations:** 1 Sport Training Lab. Faculty of Sport Sciences. University of Castilla La Mancha (Spain).; 2 Athletic Training Lab. University of Castilla La Mancha (Spain).; 3 Camilo José Cela University. Sport Science Institute, Madrid (Spain).

**Keywords:** isokinetic, handball, peak torque, power, jumping

## Abstract

Isokinetic strength evaluation of the knee flexion and extension in concentric mode of contraction is an important part of the comprehensive evaluation of athletes. The aims of this study were to evaluate the isokinetic knee peak torque in both the extension and flexion movement in the dominant and non-dominant leg, and the relationship with jumping performance. Twelve elite male handball players from the top Spanish handball division voluntary participated in the study (age 27.68 ± 4.12 years; body mass 92.89 ± 12.34 kg; body height 1.90 ± 0.05 m). The knee extensor and flexor muscle peak torque of each leg were concentrically measured at 60º/s and 180º/s with an isokinetic dynamometer. The Squat Jump and Countermovement Jump were performed on a force platform to determine power and vertical jump height. Non-significant differences were observed between legs in the isokinetic knee extension (dominant= 2.91 ± 0.53 Nm/kg vs non-dominant = 2.70 ± 0.47 Nm/kg at 60º/s; dominant = 1.90 ± 0.31 Nm/kg vs non-dominant = 1.83 ± 0.29 Nm/kg at 180º/s) and flexion peak torques (dominant = 1.76 ± 0.29 Nm/kg vs non-dominant = 1.72 ± 0.39 Nm/kg at 60º/s; dominant = 1.30 ± 0.23 Nm/kg vs non-dominant = 1.27 ± 0.35 Nm/kg at 180º/s). Low and non-significant correlation coefficients were found between the isokinetic peak torques and vertical jumping performance (SJ = 31.21 ± 4.32 cm; CMJ = 35.89 ± 4.20 cm). Similar isokinetic strength was observed between the legs; therefore, no relationship was found between the isokinetic knee flexion and extension peak torques as well as vertical jumping performance in elite handball players.

## Introduction

Handball is an Olympic team sport that requires muscular strength, power, speed, and endurance (Gorostiaga et al., 2006; Marques and González-Badillo, 2006). Handball players possess a wide range of physical skills that include throwing, diving, blocking and ball control (Wallace and Cardinale, 1997). Research in handball has focused on the seasonal changes in physical variables (Gorostiaga et al., 2006; Marques and González-Badillo, 2006), throwing velocity and strength training (Hoff and Almasbakk, 1995; Skoufas et al., 2003).

Although sporting abilities do not entail muscular contractions at a constant angular velocity (Brown and Stone, 2000), isokinetic strength has been evaluated in different sports, not only for the study of injuries (Lee et al., 2009; Eitzen et al., 2010; Cheung et al., 2012), but also to evaluate muscular torque in relation to training and performance in basketball and soccer (Zakas et al., 2005; Zakas, 2006; Bradic et al., 2009; Requena et al., 2009).

A few studies have analyzed the relationship between throwing velocity and isokinetic strength in handball (Fleck et al., 1992; Zapartidis et al., 2007). However, several studies have analysed the relationship between isokinetic knee extensor peak torque and vertical jumping performance (Genuario and Dolgener, 1980; Östenberg et al., 1998; Saliba and Hrysomallis, 2001; Tsiokanos et al., 2002; Malliou et al., 2003). Therefore, to our knowledge, no studies have been conducted to elucidate the relationship between measures of strength/power and vertical jumping in handball.

In addition, the symmetry between the dominant and non-dominant legs remains unclear, since some studies have reported the existence of symmetry in athletes, while other works have found asymmetry (Wyatt and Edwards, 1981; Jones and Bampouras, 2010; Cheung et al., 2012). These contradictory results may be due to different definitions of the “dominant leg” by various authors. Some researchers define it, as the leg preferred for kicking, others describe it as the stronger limb, and some do not specify how dominance was determined (Zakas, 2006). Side-to side strength imbalance has been suggested as a risk factor for injuries in athletes (Myer et al., 2004; Cowley et al., 2006). To the best of our knowledge, no studies have assessed the symmetry between the dominant and non-dominant legs in handball players.

Therefore, the aims of the present study were as follows: 1) to evaluate the isokinetic knee peak torque in both the extension and flexion movement in the dominant and non-dominant leg, and the jumping performance of elite male handball players; 2) to study the possible differences between legs in isokinetic peak torques; and 3) to analyse the relationship between jumping performance and isokinetic peak torques in these handball players.

## Material and Methods

### Participants

Twelve elite male handball players (age: 27.68 ± 4.12 years; body mass: 92.89 ± 12.34 kg; and body height: 1.90 ± 0.05 m) from a Spanish handball team participated in this study. These players belonged to the first division of the Spanish Handball League, one of the strongest European men’s leagues. The study was approved by the University of Castilla-La Mancha Ethics Committee, and written informed consent was obtained from the subjects.

### Measures and Procedures

The evaluations were performed in a testing session during the competitive period. Subjects performed a standardised warm-up at the start of the session, consisting of 5 minutes of cycling (Monark 818E, Varberg, Sweden) at 120 W (Bradley et al., 2007), followed by 3 minutes of rest. The participants were instructed to “cycle at a comfortable speed” (corresponding to the average cadence of 62 rpm) which was observed to be a consistent pace.

The knee extensor and flexor muscle peak torque (relative to body mass) of each leg were concentrically measured at 60º/s and 180º/s (5 repetitions each) using a Biodex System 3 isokinetic dynamometer (Biodex Corporation, Shirley, NY) according to standard procedures (Kovaleski and Heitman, 2000). Peak torque flexion-extension (flexion / extension) was also calculated.

The subject was strapped into the chair, using the lateral femoral condyle as an anatomical reference for the axis of rotation. The length of the lever arm was individually determined, and the resistance pad was placed proximal to the medial malleolus. Torque was corrected for gravity data after direct measurements of the mass of the lower limb lever arm system at 30º knee flexion (Bradic et al., 2009). The estimation of the passive torque of gravity was determined for each subject and was taken into account for the data analysis. The range of motion varied from 90º knee flexion to full extension (considering 0º as full extension). A standardized warm-up of 10 submaximal concentric contractions of the tested muscle groups at each velocity of 60º/s and 180º/s preceded the formal testing (Bradic et al., 2009). The values of the peak torques over 5 consecutive contractions for each muscle group tested were used for the data analysis. One minute of rest was allowed between assessments at different angular velocities using the protocol described by Bradic et al. (2009). All subjects indicated that their right leg was dominant (the one they would use to kick a ball). Subjects were instructed to hold their arms across the chest to isolate extension movements in the knee joint.

Vertical jumps were then performed using a force platform Kistler Quattro-Jump (Kistler, Switzerland) at 500 Hz. For the Squat Jump (SJ) the participant was instructed to adopt a squatting position (knee joint angle of 90º) and to make no countermovement. In the countermovement jump (CMJ), the subject stood in an upright position, flexed the knees and hips into a squat position and then immediately extended the knees and hips into an upward jump. The subjects’ hands were kept on their hips during both jumps. Three maximal jumps of each type were recorded, with 30 s rest between attempts. Each subject was given external encouragement throughout all the jumps. The attempt in which the highest jumping height was obtained was taken for further analysis. In addition to maximum jumping height, maximal strength and maximal power, both absolute and relative (Bosco et al., 1986), were also analysed.

### Statistical Analysis

SPSS 17.0 (SPSS Inc., Chicago, IL) was used for the statistical analysis. Mean ± standard deviations of the data were calculated. The paired t-test was carried out to analyse the differences between legs taking into account isokinetic strength variables. A Pearson correlation analysis with the Bonferroni correction was applied to study the relationships between isokinetic strength variables and the jumping height in SJ and CMJ. Statistical significance was set at p < 0.05.

## Results

Table 1 shows the variables related to the isokinetic strength obtained in the tests performed. No differences were found between the dominant and non-dominant leg in the knee extensor and flexor muscle peak torque at 60º/s or 180º/s. There were no significant differences between the dominant and non-dominant leg in peak torque flexion – extension ratio at 60º/s (dominant = 0.62 ± 0.16 *vs* non-dominant = 0.66 ± 0.22; *p* = 0.280) or 180º/s (dominant = 0.69 ± 0.16 *vs* non dominant = 0.67 ± 0.20; p = 0.322). The ratio increased significantly (p<0.001) in the movement at 180º/s from 60º/s in the dominant leg (p < 0.001) and no differences were found in the non-dominant leg.

The jumping variables are shown in Table 2. The Pearson correlation coefficients between isokinetic peak torques and jumping variables were low and non-significant (Table 3).

## Discussion

This study was performed to evaluate jumping performance and isokinetic peak torque of both knee and ankle extensors and flexors of elite male handball players in the dominant and non-dominant leg, and to analyse the relationship between jumping performance and isokinetic peak torques in these handball players.

The peak torques obtained in this study were lower than those obtained in a study with elite male basketball players (Bradic et al., 2009), and higher than in professional soccer players studied by Zakas (2006). In Bradic et al.’s work (Bradic et al., 2009) however, the tests were performed at the beginning of the preparatory period for a new basketball season, whereas in our study the measurements were carried out during the competitive period, as in the work by Zakas (2006). The results obtained in the study by Zakas et al. (2005), in which amateur soccer players performed the tests one month before the end of the competitive season, were similar to ours for the right knee extension at both 60º/s and 180º/s. The left knee extension peak torque was similar to ours at 60º/s, but lower at 180º/s. The knee flexion peak torques recorded by Zakas et al. (2005) were lower in both legs and velocities. Another work by Zakas (2006), however, examined the strength balance in the extensor and flexor muscle groups of the lower extremities in professional soccer players at different velocities (12, 60, 180 and 300º/s), with results that showed lower values than our study. Requena et al. (2009) showed similar results at 60º knee extensor peak torque, but a lower value at 180º in semi-professional soccer players measured during the final part of the pre-season period. These differences could be the result of the different time in the season (pre-season or in-season) in which the tests were performed. According to the work of Bradic et al. (2009), it is likely that changes in strength performance during the annual cycle could explain this phenomenon.

In reference to the jumping variables, jump height in the CMJ was lower than observed in Spanish second division handball players (Izquierdo et al., 2002) and lower than in other elite male handball players analysed by Gorostiaga et al. (2006). This difference could be due to variables such as the moment at which the test was carried out (end of the competitive season, or, as in our study, the middle of the competitive season), although in the work of Gorostiaga et al. (2006), the team was monitored during an entire season, and all values were higher than in our study. However, the fact that the measurement was performed using different instruments (force platform in our study *vs* contact platform in Izquierdo et al. (2002) and Gorostiaga et al. (2006)) would explain the differences found between handball players.

Non-significant differences were found between legs in the isokinetic peak torque at 60 and 180º/s in both extension and flexion. In agreement with the findings of Zakas et al. (2005) and Zakas (2006), there were no significant differences in strength balance between groups. It is possible that asymmetry might be observed when analysing the power exerted by each leg in the vertical jump; in handball, most jumps are performed with only one leg. However, the study by Zakas et al. (2005) mentioned above was conducted with soccer players, who normally have a preferred kicking leg; and no significant differences were found in the isokinetic peak torque between legs. The muscle symmetry recorded on the knee extensor and flexor muscles of players in the current study might be the result of strength training placed on the lower extremities in order to maintain similar strength on both body sides and avoid injuries caused by muscular imbalances.

With regard to the comparison of knee flexion and extension ratio (F/E), significant differences were observed between 60º/s and 180º/s. Weir et al. (1999) and Kurdak et al. (2005) reported high ratios for muscle peak torque in adolescent wrestlers for the angular velocities at 30º/s and 180º/s. F/E ratio of peak torque may provide important information about knee joint stability. The calculated F/E ratio was higher at 180º/s in the dominant leg when compared to the values of 60º/s. Explaining this imbalance for high angular velocities in the dominant leg is not possible with the present data but may be interpreted as a risk factor for muscle injury occurrence (Kurdak et al., 2005).

The absence of relationships between isokinetic strength and jumping variables disagrees with other studies which have found moderate correlations (Genuario and Dolgener, 1980; Saliba and Hrysomallis, 2001; Tsiokanos et al., 2002; Malliou et al., 2003). The majority of studies analyzed have assessed the relationships between isokinetic and jumping variables using speeds from 60 to 180º/s in isokinetic testing protocols (Genuario and Dolgener, 1980; Marques and González-Badillo, 2006; Lee et al., 2009). However, low correlations between isokinetic strength measurements and different vertical jumping tests were observed in healthy female soccer players (Östenberg et al., 1998). In the study by Malliou et al. (2003) with professional soccer players, moderate correlation coefficients were found between the knee extension at 60 and 180º/s; and the height jump at SJ and CMJ changed in relation to the training period in which they were performed. Low and non-significant correlations were found after the transition period. This indicates that the training period may be a possible factor affecting these relationships (Malliou et al., 2003).

Other aspects such as the subject’s training level and age may also affect the relationship between isokinetic peak torques and jumping performance (Malliou et al., 2003). In any case, it has been suggested (Iossifidou et al., 2005) that there are important differences in muscle activation and knee joint power development that must be taken into consideration when isokinetic tests are used to predict jumping performance. It is also necessary to consider that vertical jumping is a closed chain, multiarticular task involving stretch-shortening cycle type motion and depends largely on coordinative aspects (Bobbert and Van Ingen Schenau, 1988; Tessier et al., 2013), conversely the isokinetic exercise is an open kinetic chain using a constant angular velocity of a machine, and it is not a movement specific enough to predict jumping performance. Lack of the relationship found between jumping ability and isokinetic strength could be attributed to the inability of the activation state to increase to its maximum in the isokinetic test due to the short duration of the movement, resulting in submaximal moment production and to the muscle fibres operation at a more favourable region of their respective force-velocity relationship in the jumping action (Iossifidou et al., 2005). A limitation of our study was that the isokinetic test was performed at 60º/s and 180º/s, but not at higher velocities, which might have revealed the existence of correlations between SJ and CMJ.

The isokinetic knee extension and flexion peak torques at 60 and 180º/s in elite handball players who participated in this study were similar in both legs. Conversely, no relationship was found between isokinetic knee flexion and extension peak torques and vertical jumping performance in these players. This study provides normative data and performance standards for professional male handball players, thus enabling the conditioning coach to use this information to determine standards for the explosive and isokinetic strength of the lower extremities in the competitive season. Since all experimental tests have limitations, coaches and strength-conditioning coaches are advised to design personalised strength programmes for players with bilateral asymmetry in the knee extensor and flexor muscles. The results of this study indicate that isokinetic training is not recommended for improving jumping performance in handball and other team sport games.

## Figures and Tables

**Table 1:** Absolute (Nm) and relative (Nm/kg) isokinetic peak torques in leg muscles of elite male handball players.

	**Knee extension**				**Knee flexion**			
	**60º/s**		**180º/s**		**60º/s**		**180º/s**	

	**Absolute**	**Relative**	**Absolute**	**Relative**	**Absolute**	**Relative**	**Absolute**	**Relative**
**Dominant**	267.6 ± 46.4	2.91 ± 0.53	176.4 ± 29.6	1.90 ± 0.31	166.0 ± 44.4	1.76 ± 0.29	122.4 ± 33.9	1.30 ± 0.23

**Non-dominant**	247.2 ± 27.4	2.70 ± 0.47	168.2 ± 16.9	1.83 ± 0.29	160.4 ± 41.1	1.72 ± 0.39	119.1 ± 38.2	1.27 ± 0.35

**p**	0.139	0.162	0.442	0.475	0.372	0.406	0.381	0.395

Data are mean ± SD for 12 elite handball players.

**Table 2:** 

	**Peak force (N)**	**Relative peak force (BW)**	**Peak power (W)**	**Relative peak power (W/kg)**	**Jumping height (cm)**
**SJ**	2006.4 ± 362.3	2.31 ± 0.29	4073.5 ± 1279.7	47.18 ± 15.88	31.21 ± 4.32
**CMJ**	2348.1 ± 351.5	2.72 ± 0.45	4304.8 ± 1181.8	49.43 ± 15.96	35.89 ± 4.20

Data are mean ± SD for 12 elite handball players.

**Table 3:** Pearson correlation coefficients between isokinetic peak torques and jumping variables.

		**DOMINANT**			
		**knee extension 60°/s**	**knee flexion 180°/s**	**knee flexion 60°/s**	**knee extension 180°/s**

**CMJ height**	Pearson Correlation	0.097	0.074	−0.060	0.073
	p	0.789	0.838	0.868	0.840

**SJ height**	Pearson Correlation	−0.123	−0.111	−0.326	−0.136
	p	0.733	0.759	0.356	0.707

**CMJ Peak Power**	Pearson Correlation	−0.201	−0.003	−0.305	−0.058
	p	0.576	0.991	0.391	0.871

**SJ Peak Power**	Pearson Correlation	−0.154	0.088	−0.250	0.047
	p	0.669	0.807	0.485	0.895

		**knee extension 60°/s**	**NON-DOMINANT**		
			**knee flexion 60°/s**	**knee extension 180°/s**	**knee flexion 180°/s**

**CMJ height**	Pearson Correlation	0.150	−0.526	0.321	0.027
	p	0.679	0.118	0.364	0.938

**SJ height**	Pearson Correlation	0.175	−0.327	0.038	−0.198
	p	0.628	0.354	0.916	0.581

**CMJ Peak Power**	Pearson Correlation	−0.359	0.124	−0.341	0.103
	p	0.308	0.731	0.334	0.776

**SJ Peak Power**	Pearson Correlation	−0.433	0.184	−0.309	0.229
	p	0.211	0.609	0.384	0.522

SJ = Squat Jump, CMJ = Countermovement Jump.
